# Roller-Induced Bundling of Long Silver Nanowire Networks for Strong Interfacial Adhesion, Highly Flexible, Transparent Conductive Electrodes

**DOI:** 10.1038/s41598-017-16843-y

**Published:** 2017-11-30

**Authors:** Yan-Ren Chen, Chien-Chong Hong, Tong-Miin Liou, Kuo Chu Hwang, Tzung-Fang Guo

**Affiliations:** 1 Department of Power Mechanical Engineering, National Tsing Hua University, Hsinchu, Taiwan; 2 Department of Chemistry, National Tsing Hua University, Hsinchu, Taiwan; 3 Department of Photonics, National Cheng Kung University, Tainan, Taiwan

## Abstract

Silver nanowires (AgNWs) have been the most promising electrode materials for fabrication of flexible transparent touch panel, displays and many other electronics because of their excellent electrical properties, cost effectiveness, synthesis scalability, and suitability for mass production. Although a few literature reports have described the use of short Ag NWs in fabrication of randomly oriented Ag NW network-based electrode, their electrical conductivities are still far lower than that of Ag films. So far, no any literature report was able to provide any simple solution to fabrication of large-area and mass-manufactural ability to address the issues, such as, conductivity, transparency, electrical current withstand, bending stability, and interfacial adhesion. In the current work, we provide a simple solution to conquer the above-mentioned challenges, and report the development of long Ag NW bundle network electrodes on large area PET films that were coated, aligned, and bundled quickly and simply using a steel roller. Our developed AgNWs-bundle networks had superior performance in optoelectronic properties (sheet resistance 5.8 Ω sq^−1^; optical transmittance 89% at 550 nm wavelength), electrical current withstand up to 500 mA, and bending stability over 5000 bending cycles, and strong interfacial adhesion.

## Introduction

Transparent conductive electrodes (TCEs) with high electrical conductivity and optical transmittance are widely used in numerous optoelectronic devices, such as organic light emitting diodes, touch screens, solar cells, and wearable electronics^1–4^. Conventional TCEs are based on indium tin oxide (ITO). Thus, devices fabricated with ITO cannot be flexible, stretchable, or wearable^5,6^. Several novel materials from which flexible TCEs can be produced have been studied, including graphene^7^, carbon nanotubes^8^, hydrogels^9^, and metal nanowires^10,11^. Among these materials, silver nanowires (AgNWs) have been the most promising alternative because of their excellent electrical properties, cost effectiveness, synthesis scalability, and suitability for mass production^11,12^. In the recent years, several AgNWs problems have been partially improved. For example, because the processing temperature must be kept below 130 °C to realize TCEs on flexible polymer substrates, such as polyethylene terephthalate (PET) films, some sintering techniques other than thermal sintering have been proposed, such as a high-pressure pressing process (up to 25 Mpa)^13^, a light-induced plasmonic nanowelding technique^14^ or an electrowelding technique^15^. Although the sheet resistance of 8.6 Ω sq^−1^ with 80% optical transmittance was achieved after the above sintering process, it’s difficult to apply the above sintering techniques for large-scale TCEs process. The other critical issues, such as poor adhesion to substrates^16,17^, low bending stability (1000 cycles with a 3.1-mm bending radius)^17^, low electrical current withstand (200 mA)^15^, and poor corrosion resistance^12^, are mainly due to randomly orientated-and-distributed AgNWs structures.

Efforts have recently been made to pattern AgNW grids using neutral vapor etching^18^, inkjet printing^19^, and screen printing^20^. However, these methods result in AgNWs in each conductive trace line that are randomly oriented^11–21^, and thus do not perform optimally. In the past studies, these methods result in AgNWs on electrodes that are randomly oriented, and thus do not perform optimally for optoelectronic applications. Alignment of AgNWs are one of the ways to improve the efficiency of electrical transmission. Various techniques have been employed to align the nanowires, including the Langmuir–Blodgett technique^22^, shape memory polymer shrinking^23^, capillary printing with nanochannels^24^, water-bath assisted convective assembly^25^, and meniscus-dragging deposition^26^. Recently, 2 × 2 mm^2^ silver nanowire ordered arrays were developed by using nanotemplate imprint-lithography technique^27^. Although these techniques successfully aligned the nanowires to reach 20 Ω sq^−1^ with 92.9% optical transmittance, they required certain preparation such as additional transfer processing, substrate pretreatment, or nanoscale prepatterning, that limited the mass production ability of large devices that used them. In addition, the well-aligned NWs, which were deposited in parallel, greatly reduced the intersection points of AgNW networks. Thus, short AgNW networks with high degree of alignment lose some conductivity. In general, metal bundles have been widely used in electronics, such as in wire cords, to provide excellent electrical transmission due to the increasing of the contact area between parallel wires. Up to date, most of metal nanowire bundles are in out-of-plane structures^28,29^. Because of the difficulty of bundle manipulation and assembly of metal wires at the nano scale, only few metal nanowires have been successfully realized to in-plane bundles, such as gold nanowire bundles fabricated by using template-assisted techniques^30^.

**Figure 1:**
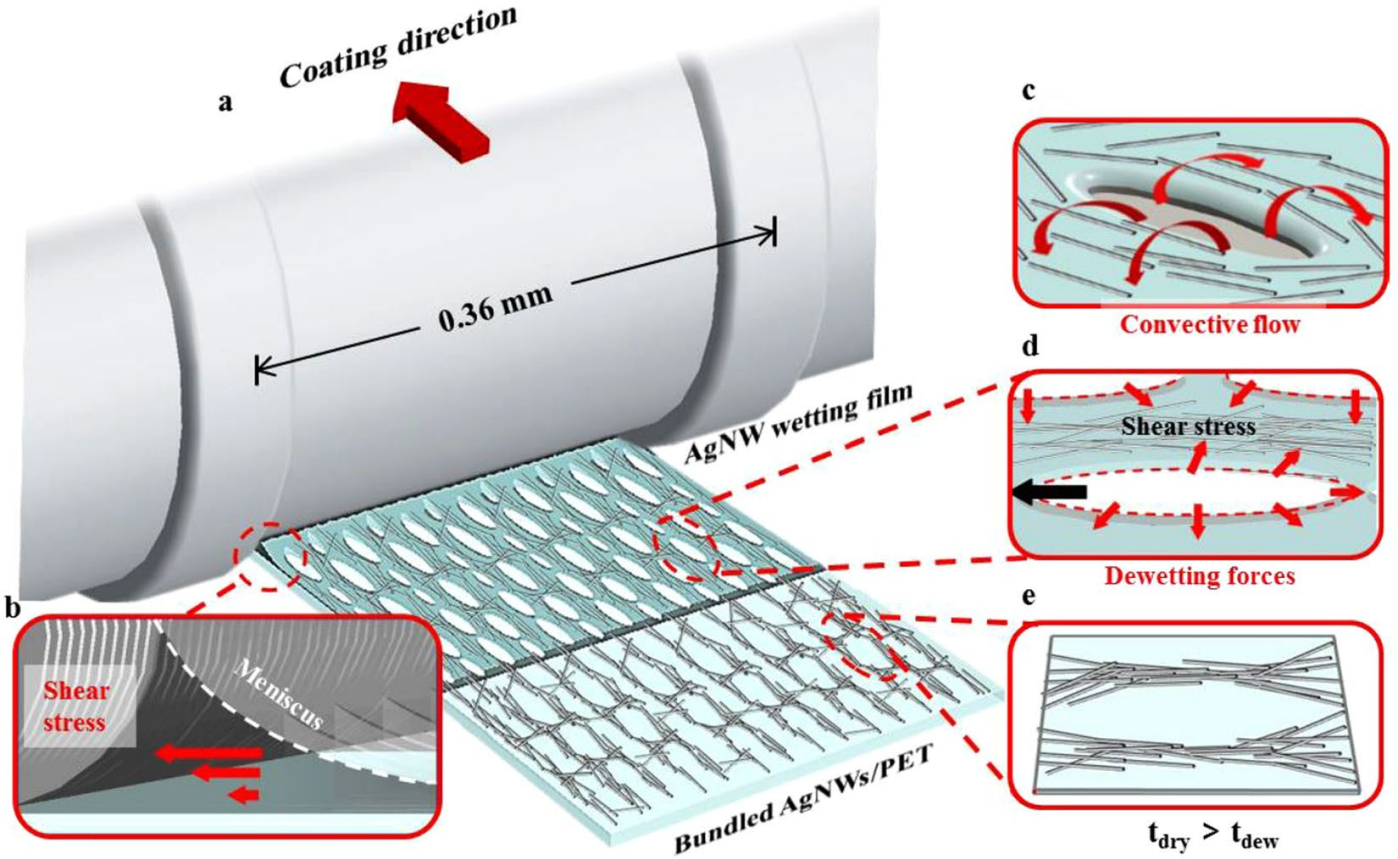
Roll coated aligned-AgNW-bundles networks. (**a**) Schematic of roll coating process to produce aligned and bundled AgNW film. (**b**) Schematic depicting the Landau-Levich meniscus which drags AgNW wetting film and causes a stress gradient exerting along the coating direction. (**c**) Schematic depicting convective flow induced by heating bought AgNWs to edge of void. (**d**) Dewetting AgNW film ruptured by dewetting force and dragged by capillary force. AgNWs in the wetting film are then squeezed into bundled AgNWs. Three stages of AgNW distribution during the wire-wound rod coating process. (**e**) AgNWs wetting film dries and the wetting film breaks simultaneously. AgNWs are squeezed into bundled AgNWs.

So far, no literatures proposed any simple solution with large-area and mass-manufactural ability to simultaneously address the AgNWs issues, such as high conductivity, high transparency, low electrical current withstand, low bending stability, and poor interfacial adhesion. In this paper, we report the development of long AgNW-bundle network electrodes on PET films that were coated, aligned, and bundled quickly and simply using a steel roller. The proposed AgNWs-bundle networks had superior performance in optoelectronic properties (sheet resistance 5.8 Ω sq^−1^; optical transmittance 89% at 550 nm wavelength), electrical current withstand (up to 500 mA),and bending stability (over 5000 bending cycles with a bending radius of 1 mm), and strong interfacial adhesion compared with ITO in the same system. Additionally, their efficiency was comparable and they greatly improved durability. This technique can be easily applied to large-scale AgNW networks, in which substantial improvement in conductivity can be achieved at low temperatures. Silver grows along the{111} facets in a polyol solution (containing PVP, NaCl and AgNO3. We synthesized AgNWs using the nitrate ion–promoted polyol process reported previously^31^, with the exception that the dispersing agent PVP with molecular weight 1300 k was used, which ensured the fabrication of high-aspect-ratio long AgNWs. After synthesis, the AgNWs were precipitated using acetone to remove the ethylene glycol (EG) in the original solution. The AgNWs were then resuspended with isopropyl alcohol (IPA) to a concentration of 20 mg mL^−1^. AgNWs of three lengths and similar diameters were obtained for the experiments that followed. Synthesized AgNWs with length 82 μm and diameter 45 nm had the highest aspect ratio (up to 1800), as displayed in Figure S1a (Supporting Information). Single AgNW was measured about 4.95 Ω/μm. We prepared aligned AgNW-bundle networks through a rolling process (Fig. 1a), which coated PET films quickly and easily. AgNW networks were rolled onto 125-μm PET films using a Meyer rod #14 wound with wire of diameter 0.36 mm, and were heated on a hotplate at 120 °C. The AgNW network–coated PET film was rinsed with DI water for 30 s to remove PVP from AgNW surfaces and then dried on a hotplate. The aligning and bundling process was dominated by the following mechanisms: (i) The Landau–Levich meniscus dragged the wetting film and created a sheer stress gradient in the coating direction (Fig. 1b)^32^. (ii) Evaporation-induced convective flow carried the AgNWs into the dry voids formed by surface tension (Fig. 1c). (iii) As the AgNW wetting film dried out, AgNWs were squeezed between two voids and aligned AgNW-bundle networks were formed. The deposited AgNW bundles had an elliptical shape (Fig. 1d).

**Figure 2:**
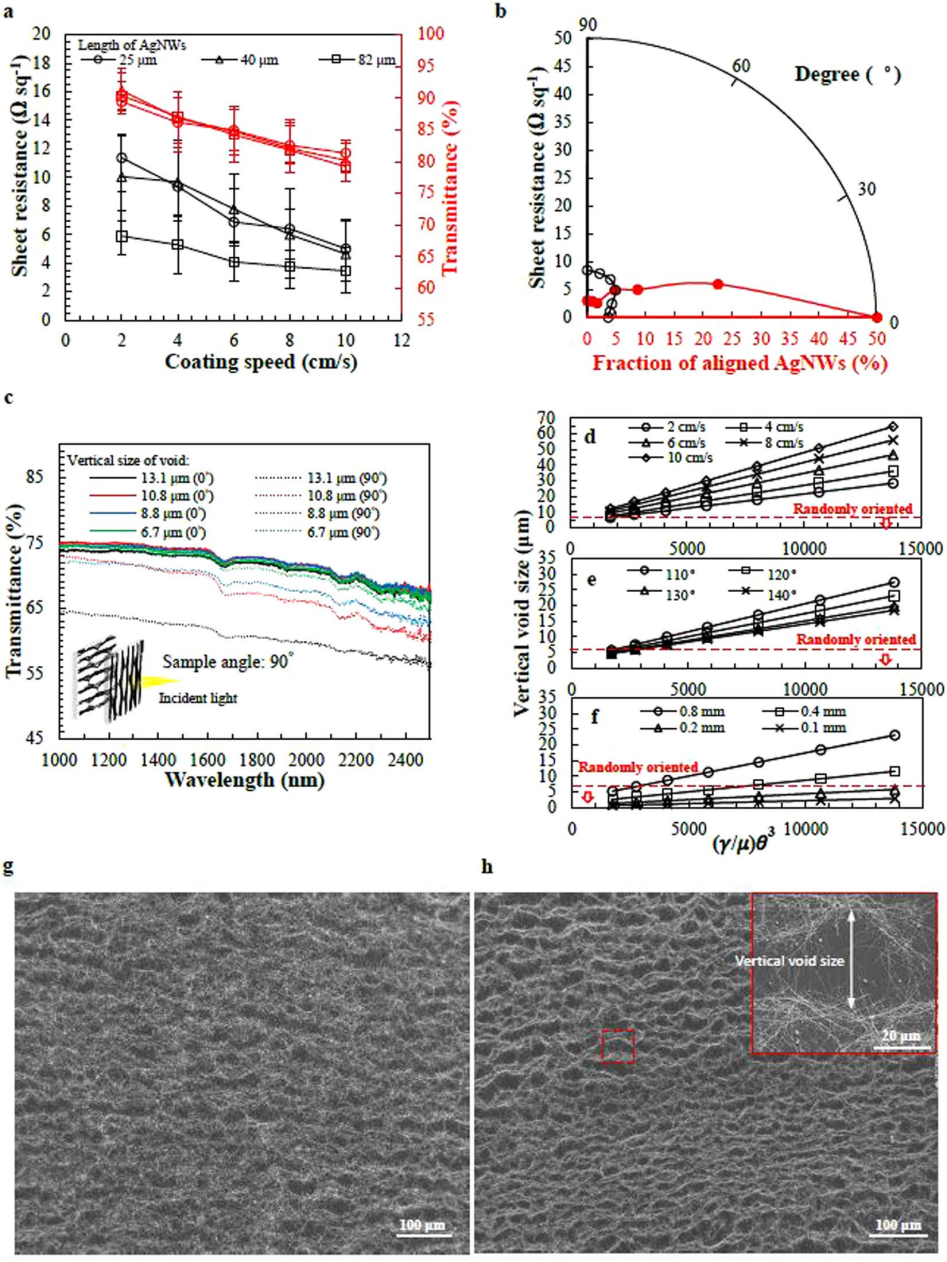
Characterization of aligned-AgNW-bundles networks. (**a**) The performance of three different lengths of AgNWs at different coating speeds. Sizes of AgNWs are depicting in Figure S1a. (**b**) Polar plots of angles versus the sheet resistance and the fraction of aligned AgNWs when coating speed is 10 cm/s. (**c**) NIP transmittance of aligned-AgNW-bundles network with different void size. (Inset) two aligned-AgNW-bundles films oriented perpendicular for NIR transmittance measurement. (**d**) Calculated results of void size versus AgNWs suspension properties from coating speed 2 cm/s to 10 cm/s. The results were calculated when drying temperature is 120 °C and the gap height is 0.8 mm. (**e**) Calculated results of void size versus AgNWs properties under drying temperatures from 110 °C to 140 °C. The results were calculated with a coating speed of 2 cm/s and a gap height of 0.8 mm. (**f**) Calculated results of void size versus AgNWs properties with gap height 0.8 mm to 0.1 mm. The results were calculated with a coating speed of 2 cm/s and a drying temperature of 120 °C. SEM image of aligned AgNW bundles network for a coating speed of (**g**) 4 cm/s and (**h**) 10 cm/s.

In conventional roll coating, the wet film dries before it breaks^11,12,33^. In our approach, however, we dried the AgNW wetting film more slowly, over a period nearly equal to the time it would take the AgNW wetting film to dry naturally. AgNW-bundle networks were formed by controlling the drying temperature and rolling speed simultaneously. The capillary fluid in the Landau–Levich meniscus near the substrate induced a shear stress that prolonged the voids of the AgNW wetting film during drying. The film’s thickness was calculated using the following equation: h = $\,1.34{(\frac{\mu U}{\gamma })}^{2/3}{\rm{r}}$, where *μ* is the viscosity of the AgNW suspension, U is coating speed, *γ* is surface tension, and r is the meniscus radius. Due to the small wire diameter (0.36 mm) on the roller, which induces meniscus flow, the radius of meniscus can be simplified to r = 2*h*_0_, where *h*_0_ represented gap height between the roller and the PET substrate^34^. If the coating speed is increased, the viscous suspension stretches the meniscus and increases its radius, leading to a thicker wetting film. The properties of AgNW suspensions are listed in Table 1, and the relationship between thickness and coating speed is calculated and displayed in Figure S1b. Figure 2a depicts the measured sheet resistance when different coating speeds were used. Evaporation-induced convective flow and surface tension–induced flow are the two major factors that cause the wetting film breakage and subsequent squeezing and merging of AgNWs into bundles. The void sizes caused by the convective flow, named the Marangoni flow, are calculated as $\,{\rm{\lambda }}=\frac{1}{2}{\rm{\pi }}h\sqrt{\frac{{M}_{a}}{32}}$, where M_a_ is the Marangoni number, h is the wetting film thickness, and λ is half of the void size^35^. The fluid system is dominated by evaporation-induced convective flow when M_a_ is larger than 80. As mentioned, the AgNW wetting film is thicker if a higher coating speed is used, and the sheet resistance of the aligned AgNW-bundle network is lower. Using this approach, we can control the sheet resistance not only by tuning the AgNW suspension concentration, but also by adjusting the coating speed.

**Table 1:** List of physical properties and coating related numbers of AgNW suspension with different length and heating duration.

Length (μm)	Heating duration (hours)	Concentration (mg/mL)	Viscosity (mPa·s)	Surface tension (mN/m)	Contact angle (°)	${\boldsymbol{(}}{\boldsymbol{\gamma }}{\boldsymbol{/}}{\boldsymbol{\mu }}{\boldsymbol{)}}{{\boldsymbol{\theta }}}^{{\rm{3}}}$	Film thickness (μm) at different coating speed (calculated)				
							2 cm/s	4 cm/s	6 cm/s	8 cm/s	10 cm/s
25	4	25.1	21.9	22.6	15.3°	3470	1.54	2.45	3.21	3.89	4.52
40	8	24.3	21.4	21.5	14.9°	3292	1.57	2.51	3.27	3.97	4.61
82	12	25.2	22.8	21.7	15°	3546	1.63	2.59	3.40	4.12	4.78

The dewetting velocity of the AgNW suspension was calculated by ${V}_{dewet}=(\tfrac{k\sigma }{\mu }){\theta }^{3}$ ^33^, where k is a property of the fluid and can be taken as 10^−6^ for an IPA-based system. We calculated the dewetting and drying velocities to be 918 and 106 μm s^−1^, respectively. The dewetting and drying duration were calculated by dividing the wetting film thickness by the dewetting and drying velocity. Combining the evaporation-induced and surface tension–induced flow, the void sizes of the wetting film during coating are given by the following equation: Hole size $={\rm{E}}{\rm{\pi }}{{\rm{h}}}^{2}{\rm{B}}\bigtriangleup {\rm{T}}+{\rm{S}}{\rm{\pi }}{\rm{h}}\gamma {\theta }^{3}$. ${\rm{E}}=\,\sqrt{\frac{1}{128\rho \mu \alpha }}$ and ${\rm{S}}=\,\frac{k}{\mu {J}_{o}}$ are the viscosity-related numbers of the AgNW suspension, and B is the surface tension gradient caused by the temperature gradient $\bigtriangleup {\rm{T}}$. The thickness of the wetting film increased from 1.63 to 4.78 μm when the coating speed was increased from 2 to 10 cm s^−1^, as calculated and shown in Figure S1b. Calculated and measured vertical and horizontal void sizes are compared in Figure S1c, d. As the wetting film thickness increased because of an increase in coating speed, the voids grew larger because of a longer drying time.

The AgNWs were squeezed into aligned bundle networks dominated by surface tension effects. We first examined the alignment directions of the AgNWs bundles and the void sizes between AgNW bundles when various coating speeds were used, as shown in Figures S2a–f. The voids produced were larger if a higher coating speed was used, and the experimental results were in agreement with the calculated results. The AgNW-bundle network fabricated using a coating speed of 10 cm s^−1^ was the most aligned. Scanning electron microscopy (SEM) images of bundled AgNWs fabricatsed using various coating speeds are displayed in Fig. 2g, h and S3. Figure 2b is a polar plot of the sheet resistance and the fraction of AgNWs that were aligned when a coating speed of 10 cm s^−1^ was used. From the measurements, more than 85% of AgNWs in the network were oriented within ± 30° of the average orientation for this speed. The correspondence between vertical void size and coating speed, as shown in Figure S4. The sheet resistances parallel and perpendicular to the coating direction were 3.6 Ω sq^−1^ and 8.5 Ω sq^−1^, respectively. This electrical anisotropy was due to the weak connectivity of the aligned AgNWs perpendicular to the coating direction. Figure 2c shows the polarized transmittance at NIR spectrum of aligned-bundles AgNWs network with vertical void sizes from 13.1 μm to 6.7 μm. The spectrum from wavelength 1000 nm to 2500 nm were obtained at polarization angle of 0° and 90° that represent two sample located parallel and perpendicular, respectively, under an un-polarized light source. The polarized transmittance demonstrated the prominent anisotropies of aligned-bundles AgNWs network. The transmittance of AgNWs network with void size 13.1 μm observed at polarization angle of 0° is 10% higher than that at polarization angle of 90° (8.3% degree of polarization at 2500 nm). For the void size of AgNWs network keeping narrowing down to the wavelength of NIR, the degree of polarization should enhance according to Saito’s equation. However, as the void size keeping narrowing down, the degree of alignment decreased as well. As a result, the degree of polarization dropped with decreasing void size. For AgNWs network with average void size 6.7 μm, the transmittance at NIR spectrum shows 2% difference between polarization angle of 0° and 90° (1.5% degree of polarization at 2500 nm). Hence, we defined align-bundles AgNWs network with void size 6.7 μm were randomly oriented. Figure 2d–f depicts the calculated void size under different conditions of roll coating process versus properties of AgNWs/IPA suspension to fabricate align-bundles AgNWs network. AgNWs suspension properties on PET substrate $(\frac{\sigma }{\mu }){\theta }^{3}$ represented the dewetting velocity of wetting film. For Fig. 2d, the connection between coating speed and void size is shown and calculated with a drying temperature of 120 °C and a gap height of 0.8 mm. It is notable that void size of AgNWs network below 6.7 μm was defined randomly oriented in this paper and void size above 40 μm could be regarded as AgNWs tangled during the roll coating process. As for Fig. 2e, the connection between drying temperature and void size is shown with 2 cm/s coating and 0.8 mm gap height. By tuning the roll coating conditions, we can easily align and bundle AgNWs and obtain aligned-bundles AgNWs network with different void sizes. Figure 2f demonstrates the relationship between gap height and AgNWs suspension properties on the PET substrate. Reported roll coating process to date was operating at condition that the roller was closely contacted with target substrate (gap height < 0.1 mm). Here, we improve the roll coating process that prominently aligned and bundled long AgNWs. The mechanisms of dewetting and evaporation induced aligning and bundling process and degree of AgNWs alignment were fully development. From the results, the regions for roll-coated randomly oriented AgNWs network and aligned-bundles AgNWs network are calculated and determined in Fig. 2d–f.

**Figure 3:**
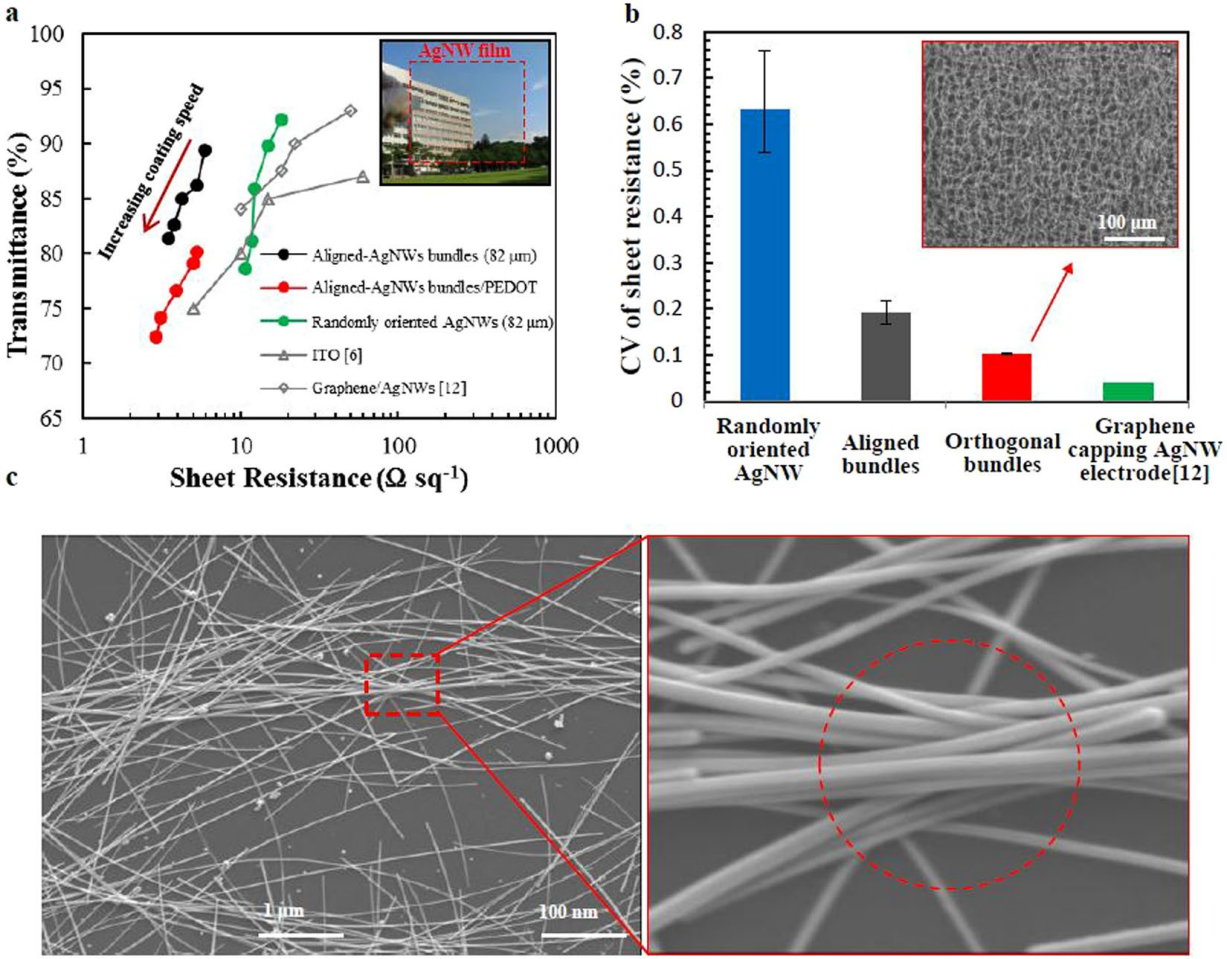
Characterization of aligned –AgNW-bundles networks as transparent conductive electrodes. (**a**) Sheet resistance versus transmittance (at 550 nm) for electrodes assembled with three different lengths of AgNWs. (**b**) Uniformity of randomly oriented AgNW, aligned and bundled AgNW, aligned, bundled, and orthogonal AgNW electrodes, and graphene/AgNW hybrid electrode. (Inset) SEM image of orthogonal-aligned AgNW bundles network. (**c**) SEM image of a bundle node, which was formed by squeezing AgNWs with surface tension.

PVP on AgNW surfaces, which causes a high contact resistance of up to 1000 Ω sq^−1^ between AgNWs, can be removed by water rinsing^14,36^. For a randomly oriented AgNW network without bundling, the sheet resistance was found to be 14.8 Ω sq^−1^ with 89% optical transmittance (at 550 nm). After water rinsing was performed, the AgNW-bundle networks assembled using AgNWs of length 82 μm performed outstandingly, having a sheet resistance and optical transmittance of 5.8 Ω sq^−1^ and 89% for a coating speed of 2 cm s^−1^, and 3.4 Ω sq^−1^ and 81% for a coating speed of 10 cm s^−1^, respectively, that are superior to those found when ITO^6^ and other AgNW-based hybrid electrodes^24^ were used, as illustrated in Fig. 3a. The sheet resistance of the AgNW networks was higher when coating speed was increased, but the optical transmittance decreased. Compared with graphene–Ag nanowires^12^ and randomly oriented AgNW networks, the sheet resistance of aligned AgNW-bundle networks varied little at 0.19% and 0.1% for the aligned and orthogonal bundles, respectively, as shown in Fig. 3b. Figure 3c shows the bundle and the bundle node, which boosted the performance in electronics and mechanics.

**Figure 4:**
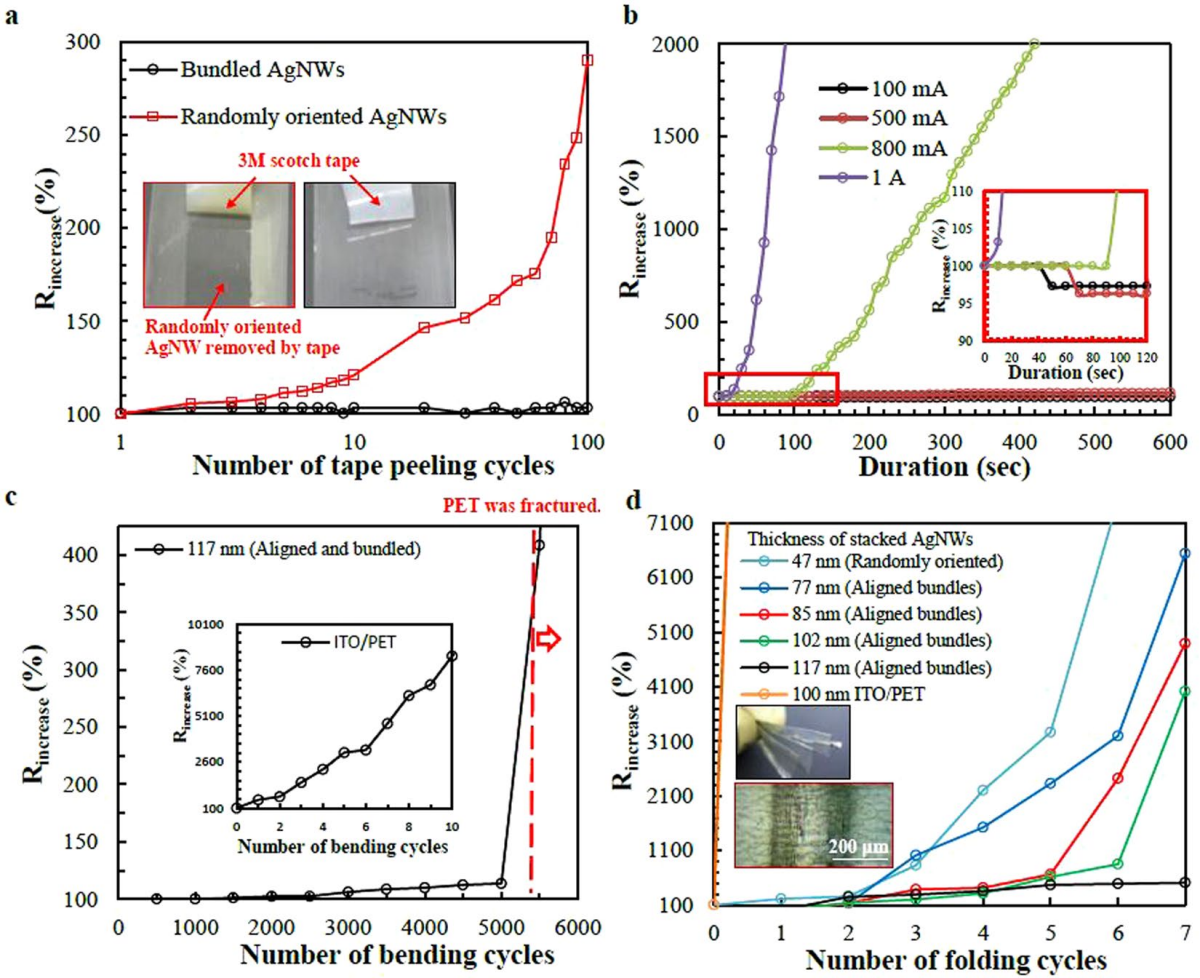
Flexibility and durability of AgNW electrodes with different thickness. (**a**) Tape peeling cycles for randomly oriented AgNWs and aligned-AgNWs-bundles electrodes. (**b**) Sheet resistance changes with time under different currents. (Inset) The sheet resistance changes at the very beginning. (**c**) Bending cycles of aligned-AgNW-bundles networks and (Inset) ITO/PET. (**d**) Folding cycles for aligned-AgNW-bundles electrodes. (Inset) The picture (upper) and optic microscope image (lower) of AgNW electrode after the folding.

The sheet resistance increased 300% after the randomly oriented AgNW network without bundling was peeled with a 3 M scotch tape 100 times. However, in addition to having boosted electrical conductance, the aligned AgNW-bundle network after PVP removal also demonstrated strong adhesion to the PET substrate, and had the same sheet resistance after 100 peelings, as depicted in Fig. 4a. The strong adhesion to the PET substrate was probably due to the AgNW-bundle nodes. Firm adhesion to the substrate can improve the bending performance of the AgNW network because most failures are caused by loose contact between AgNWs and the substrate^16,17^. In addition to have stronger adhesion, bundled AgNWs can survive post-treatments or subsequent processes such as polymer spin coating or solvent cleaning that are used in the fabrication of optoelectronic devices.

Corrosion resistance and stability under harsh conditions are critical concerns for metal nanowire-based TCEs^22,23,37^. Flexible polymer substrates such as PET are porous; therefore, vapor, oxygen, and sulfide gases that can damage AgNWs can penetrate PET with sufficient time. Furthermore, optoelectronic devices are designed for operation under temperatures that fluctuate regularly and substantially. Hence, thermal fatigue stability is essential for their practical applications. AgNW-bundle networks of various thicknesses and randomly oriented AgNW networks were heated for 30 min and then allowed to cool at room temperature for 5 min to measure the sheet resistance. The AgNW networks were operated at 200 °C and the cycle was repeated 48 times on glass substrates, as shown in Figure S5. The SEM images in Figure S5 (inset), taken after the 48 thermal fatigue cycles, show AgNW bundles (upper) and randomly oriented AgNWs (lower). The sheet resistance of the bundled AgNWs of thickness 117 nm increased by 228%, whereas that of the randomly oriented AgNWs increased by more than 1000%. To explain why the durability of randomly oriented AgNWs without bundling and AgNW bundles differs, we checked the morphology of the electrodes using the SEM images. The randomly oriented AgNWs were severely oxidized and had broken apart, which caused their extreme sheet resistance. The morphology of the AgNW bundles, however, remained unchanged after 48 cycles at 200 °C. AgNWs were also observed under applied electrical currents, as shown in Fig. 4b. The randomly oriented AgNW network was damaged at 0.1 A and the sheet resistance of the AgNW-bundle network slightly decreased 2.7% under an applied current of 500 mA.

Transparent conductive layers on flexible plastic films suffer from mechanical fracture mainly during repeated bending when used in flexible optoelectronics. Some studies have performed bending reliability tests consisting of 1000 bends with a radius of 1.25 mm for the aligned AgNWs electrodes^24^ and 3000 bends with a bending radius of 5 mm for the AgNWs/PEDOT/ITO composite electrodes^37^. The bending reliability of our AgNW network TCEs was tested by bending them more than 5000 times with a bending radius of 1 mm, and only an 13% change in resistance after 5000 times was demonstrated until the PET film fractured before the 5500^th^ cycles, as shown in Fig. 4c. When a PET film was coated with ITO, resistance increased by 550% after only one bending, as shown in Inset of Fig. 4c. The AgNW-bundle networks with thickness of 117 nm remained highly conductive after seven full folds, as shown in Fig. 4d. We estimated that the enhancement of strength against folding could be a result of the AgNW bundling. Furthermore, AgNWs with larger stacked thicknesses were stronger against folding.

Bulk heterojunction photovoltaic cells using the P3HT:PCBM blend were fabricated with ITO and aligned AgNW-bundle networks on PET films capped with a layer of PEDOT:PSS, as shown in Figure S6a and b. ITO/PET and the aligned AgNW-bundle network on PET had similar characteristics, with open circuit voltages of 358 and 310 mV, short circuit currents of 18.69 and 18.56 mA cm^−2^, fill factors of 0.30 and 0.32, and power conversion efficiencies of 2.01% and 1.84%, respectively. The surface roughness of the aligned AgNW-bundle networks was 104 nm, and this was pressed to 66 nm. After PEDOT:PSS capping, the surface roughness was further reduced to 14 nm, which is close to that of ITO/PET, and resulted in a similar shunt resistance of 413 Ω compared with 459 Ω, respectively. Current density versus voltage for cells under 100 mW cm^−2^ AM1.5 G illumination is shown in Figure S6c. Our AgNW-bundle network–based solar cell achieved 91.5% of the power conversion efficiency of ITO on PET films. The low sheet resistance and high transmittance of the AgNW-bundle networks resulted in highly efficient carrier transport and a reduction in the power lost within the networks.

We demonstrate transparent conductive AgNW networks, which the long AgNW nanowires were manipulated to arrange in bundles caused by evaporation-and-surface-tension-induced convective flow during the roll coating process. We tuned the performance, mechanical properties, and stability of the network through the control of the coating speed and substrate temperature. The polarization measurements have the same tendency as the morphological measurements from the SEM images. Compared with the randomly oriented-and-distributed nanowires, the nanowires, which are bundled by the roller, are redistributed on the plastic films to slightly improve optical transmittance but are greatly enhanced for conductivity, flexibility, and interfacial adhesion. The boosted performance of the developed AgNW networks, which is due to the bundling of the long nanowires, is comparable or even superior to other TCEs, such as ITO, graphene^7^, aligned AgNW networks^24^, and AgNWs/PEDOT/ITO^37^, as shown in Table 2. This simplicity of this approach can be further extended to other metal nanowire networks, such as gold, copper, and aluminum. Our aligned AgNW-bundle networks, which demonstrated boosted performance and remarkable advantages over other networks, are suitable as electrodes for flexible transparent electronic devices.

**Table 2:** List of transparent conductive electrode performances with different materials and composition. Transmittance is compared at 550 nm and the uniformity is presented with coefficient of variation, which were measured with TCEs sheet sizes 0.6 × 0.6 cm^2^ to 1 × 1 cm^2^. The adhesion was tested by 3 M scotch tape.

Materials on PET	Sheet resistance (Ω/ sq^−1^)	Transmittance (%)	Uniformity (%)	Adhesion		Flexibility			R2R
				Tape peeling	R_increase_	Radius (mm)	bending cycles	R_increase_	
ITO^6^	15	85	~0.013	100	100%		X		O
Graphene^7^	12	90	~0.05	N/A		X	X	X	O
AgNWs^10^	8	80	N/A	100	N/A	X	X	120%	O
Graphene/AgNWs^12^	8	94	0.04	100	100%	20	1000	120%	O
AgNWs^15^	19.7	86	N/A	N/A		X	X	X	△
Aligned AgNWs^24^	19.5	96.7	N/A	N/A		1.25	1000	100%	△
AgNWs/PEDOT/ITO^37^	44	88	N/A	N/A		5	3000	100%	O
Aligned–AgNWs bundles (This work)	5.88	89.4	0.1~0.19	100	103%	1	5000	113%	O

## Experimental Section

### Sources of chemicals

EG (J.T. Baker), silver nitrate (AgNO3, Showa), poly(vinylpyrrolidone) (PVP, Aldrich, MW = 1300 k), sodium chloride (Showa), and potassium nitrate (Showa) were used as received.

### Silver nanowire synthesis

NaCl (50 μL, 0.766 M in water) was added to an EG solution (10 mL) in a glass vial, followed by the addition of KNO_3_ (1 g). The solution was then maintained at 140 °C in an oil bath and stirred constantly at 50 rpm until all the KNO_3_ dissolved. Another EG solution (10 mL) containing PVP (0.396 M in terms of repeating monomer units) and AgNO_3_ (0.15 g) was mixed and then slowly injected into the 140 °C EG solution. The final solution was then heated at 140 °C for an additional 4, 8, and 12 h in an aerobic environment. The AgNW products were injected into acetone and then precipitated to remove the remaining EG. Next, the acetone was removed and the precipitated AgNWs were blowdried with nitrogen. Finally, the AgNWs were redispersed in isopropyl alcohol (10 mL), resulting in a AgNW suspension of concentration 20 mg mL^−1^.

### Electrode fabrication

The 125 μm optical PET substrate (General Hong Co.) with a contact angle of 2.71° for IPA droplets was fixed on a heated flat aluminum plate by 3 M scotch tape. AgNW suspension was then aligned and bundled through a Meyer rod #14 with wire diameter 0.36 mm (RD Specialist Inc.) at 120 °C. Aligned AgNW-bundle networks were then immersed in water for 30 s to remove the PVP coating.

### Electrode and device characterization

The optical transmittance spectra of the films were measured using a UV-vis spectrometer (JASCO V630). The surface morphology of the AgNWs was analyzed using SEM (JSM-7000F) and a surface morphology scanner (BRUKER DektakXT). The sheet resistances of the films were measured using a four-point probe system (AGILENT B52902A).

## Electronic supplementary material

Supplementary Information

## References

[1] Lee J (2013). Room-temperature nanosoldering of a very long metal nanowire network by conducting-polymer-assisted joining for a flexible touch-panel application. Adv. Funct. Mater..

[2] Liang J, Li L, Niu X, Yu Z, Pei Q (2013). Elastomeric polymer light-emitting devices and displays. Nat. Photonics.

[3] Wu C, Kim TW, Li F, Guo T (2016). Wearable electricity generators fabricated utilizing transparent electronic textiles based on polyester/Ag nanowires/graphene core−shell nanocomposites. ACS Nano.

[4] Gaynor W, Burkhard GF, McGehee MD, Peumans P (2011). Smooth nanowire/polymer composite transparent electrodes. Adv. Mater..

[5] Kumar A, Zhou C (2010). The race to replace tin-doped indium oxide: which material will win?. ACS Nano.

[6] Ellmer K (2012). Past achievements and future challenges in the development of optically transparent electrodes. Nat. Photonics.

[7] Bae S (2010). Roll-to-roll production of 30-inch graphene films for transparent electrodes. Nat. Nanotechnol..

[8] Tenent RC (2009). Ultrasmooth, large-area, high-uniformity, conductive transparent single-walled-carbon-nanotube films for photovoltaics produced by ultrasonic spraying. Adv. Mater..

[9] Kim CC, Lee HH, Oh KH, Sun JY (2016). Highly stretchable, transparent ionic touch panel. Science.

[10] Hu L, Kim HS, Lee JY, Peumans P, Cui Y (2010). Scalable coating and properties of transparent, flexible, silver nanowire electrodes. ACS Nano.

[11] Groep JVD, Spinelli P, Polman A (2012). Transparent conducting silver nanowire networks. Nano Lett..

[12] Deng B (2015). Roll-to-roll encapsulation of metal nanowires between graphene and plastic substrate for high-performance flexible transparent electrodes. Nano Lett..

[13] Tokuno T (2011). Fabrication of silver nanowire transparent electrodes at room temperature. Nano Res..

[14] Garnett EC (2012). Self-limited plasmonic welding of silver nanowire junctions. Nat. Mater..

[15] Song TB (2014). Nanoscale joule heating and electromigration enhanced ripening of silver nanowire contacts. ACS Nano.

[16] Sun Q (2015). Positively-charged reduced graphene oxide as an adhesion promoter for preparing a highly-stable silver nanowire film. Nanoscale.

[17] Jin Y, Deng D, Cheng Y, Kong L, Xiao F (2014). Annealing-free and strongly adhesive silver nanowire networks with long-term reliability by introduction of a nonconductive and biocompatible polymer binder. Nanoscale.

[18] Wu J (2016). Multi-length scaled silver nanowire grid for application in efficient organic solar cells. Adv. Funct. Mater..

[19] Finn DJ, Lotya M, Coleman JN (2015). Inkjet printing of silver nanowire networks. ACS Appl. Mater. Interfaces.

[20] Liang J, Tong K, Pei Q (2016). A Water-based silver-nanowire screen-print ink for the fabrication of stretchable conductors and wearable thin-film transistors. Adv. Mater..

[21] Kim S, Kim SY, Chung MH, Kim J, Kim JH (2015). A one-step roll-to-roll process of stable AgNW/ PEDOT:PSS solution using imidazole as a mild base for highly conductive and transparent films: optimizations and mechanisms. J. Mater. Chem. C.

[22] Tao A (2003). Langmuir−Blodgett silver nanowire monolayers for molecular sensing using surface-enhanced Raman spectroscopy. Nano Lett..

[23] Bang J (2014). Assembly and densification of nanowire arrays via shrinkage. Nano Lett..

[24] Kang S (2015). Capillary printing of highly aligned silver nanowire transparent electrodes for high-performance optoelectronic devices. Nano Lett..

[25] Duan SK (2015). Water-bath assisted convective assembly of aligned silver nanowire films for transparent electrodes. Phys. Chem. Chem. Phys..

[26] Ko Y, Song SK, Kim NH, Chang SH (2016). Highly transparent and stretchable conductors based on a directional arrangement of silver nanowires by a microliter-scale solution process. Langmuir.

[27] Sciacca B, Groep JVD, Polman A, Garnett EC (2016). Solution-grown silver nanowire ordered arrays as transparent electrodes. Adv. Mater..

[28] Wang R, Ruan C, Kanayeva D, Lassiter K, Li Y (2008). TiO_2_ nanowire bundle microelectrode based impedance immunosensor for rapid and sensitive detection of Listeria monocytogenes. Nano Lett..

[29] Zhu C (2016). A hierarchically ordered array of silver-nanorod bundles for surface-enhanced Raman scattering detection of phenolic pollutants. Adv. Mater..

[30] Maurer JHM, González-García L, Reiser B, Kanelidis I, Kraus T (2016). Templated self-assembly of ultrathin gold nanowires by nanoimprinting for transparent flexible electronics. Nano Lett..

[31] Kuo CL, Hwang KC (2012). Nitrate ion promoted formation of Ag nanowires in polyol processes: a new nanowire growth mechanism. Langmuir.

[32] Park B, Bae IG, Huh YH (2016). Aligned silver nanowire-based transparent electrodes for engineering polarisation-selective optoelectronics. Scientific Reports.

[33] Dan B, Irvin GC, Pasquali M (2009). Continuous and scalable fabrication of transparent conducting carbon nanotube films. ACS Nano.

[34] Jeon HG, Cho CY, Shin JC, Park B (2012). Inverted polymer solar cells fabricated by a pre-metered coating process. Journal of Materials Chemistry.

[35] Stannard A (2011). Dewetting-mediated pattern formation in nanoparticle assemblies. Phys.: Condens. Matter.

[36] Wang J (2015). Silver nanowire electrodes: conductivity improvement without post-treatment and application in capacitive pressure sensors. Nano-Micro Lett..

[37] Yoo JH (2015). Silver nanowire−conducting polymer−ITO hybrids for flexible and transparent conductive electrodes with excellent durability. ACS Appl. Mater. Interfaces.

