# Supplementary material for: Roller-Induced Bundling of Long Silver Nanowire Networks for Strong Interfacial Adhesion, Highly Flexible, Transparent Conductive Electrodes
Source: Sci Rep. 2017 Nov 30;7:16662. doi: 10.1038/s41598-017-16843-y (PMC5709471; doi:10.1038/s41598-017-16843-y)
Supplement: Supplementary file 1 — Supplementary Information [file 41598_2017_16843_MOESM1_ESM.pdf]

Roller-Induced Bundling of Long Silver Nanowire Networks for Strong Interfacial Adhesion, Highly Flexible, Transparent Conductive Electrodes

Yan-Ren Chen[1], Chien-Chong Hong*[1], Tong-Miin Liou*[1], Kuo Chu Hwang[2], and Tzung-Fang Guo[3]

[1]Department of Power Mechanical Engineering, National Tsing Hua University, Hsinchu, Taiwan,

[2]Department of Chemistry, National Tsing Hua University, Hsinchu, Taiwan

[3]Department of Photonics, National Cheng Kung University, Tainan, Taiwan

Corresponding authors:

Prof. Chien-Chong Hong, Tel: 886-3-5715131ext.33736, Fax: 886-3-5722840,

email: chong@pme.nthu.edu.tw,

Prof. Tong-Miin Liou, Tel: 886-3-5715131ext.42607, Fax: 886-3-5722840,

email: tmliou@pme.nthu.edu.tw

address: Department of Power Mechanical Engineering, National Tsing Hua University, 101, Sec. 2, Kuang Fu Rd., Hsinchu, Taiwan

Supporting information

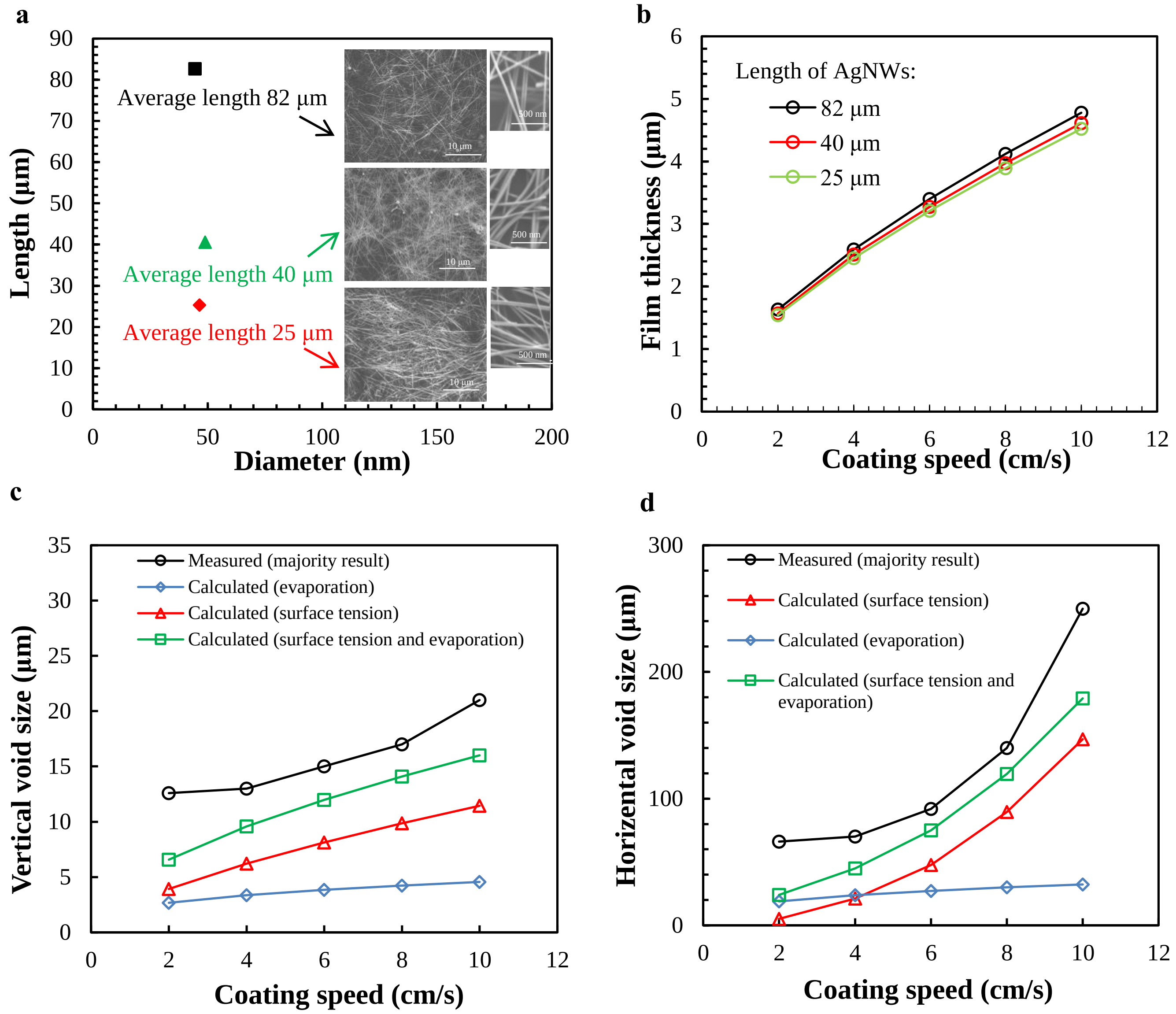

Figure S1. (a) Lengths and diameters of AgNWs with a heating duration of 4, 8, 12 hours which correspond to lengths of 82, 40, 25 μm, respectively. (Inset) SEM image of AgNWs with various heating durations. (b) Calculated wetting film thickness of different AgNWs with different heating durations and coating speeds. (c) Comparisons between calculated vertical void sizes of evaporation, surface tension induced hollows and measured results. (d) Comparisons between calculated horizontal void sizes of evaporation, surface tension induced hollows and measured results.

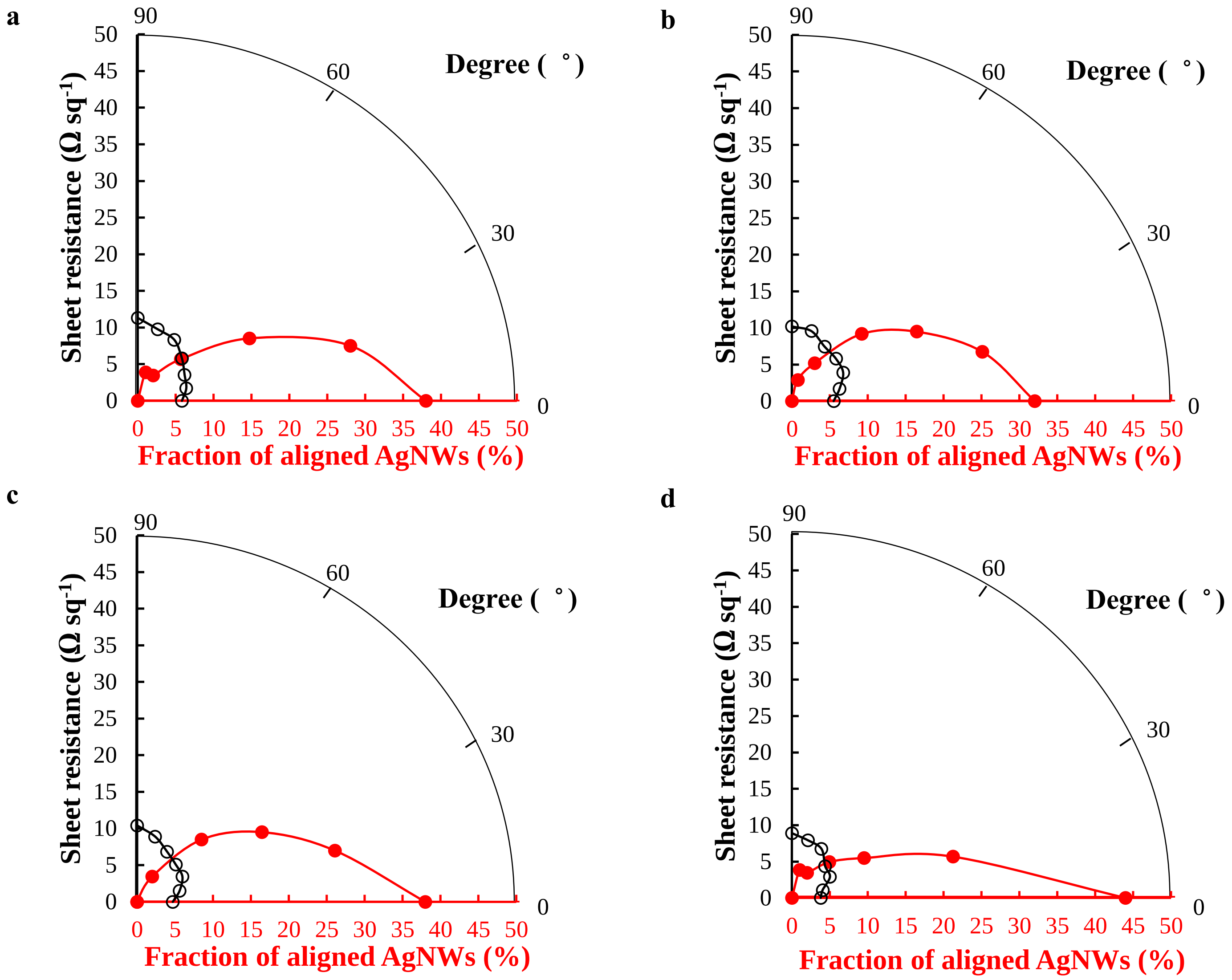

Figure S2. Polar plots of angles versus the sheet resistance and fraction of aligned AgNWs for a coating speed of (a) 2, (b) 4, (c) 6, and (d) 8 cm s^{-1}.

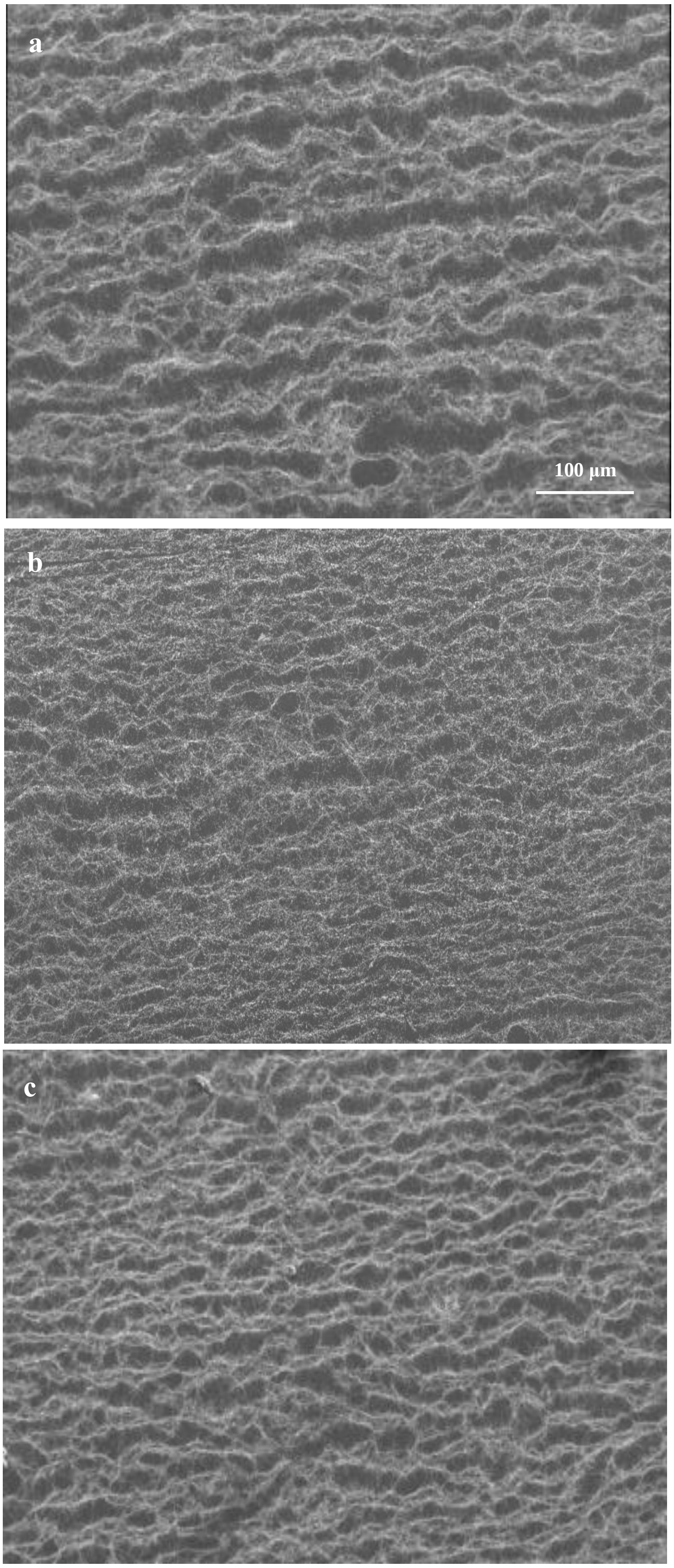

Figure S3. SEM image of aligned AgNW bundles network for a coating speed of (a) 2, (b) 6, and (c) 8 cm s^{-1}.

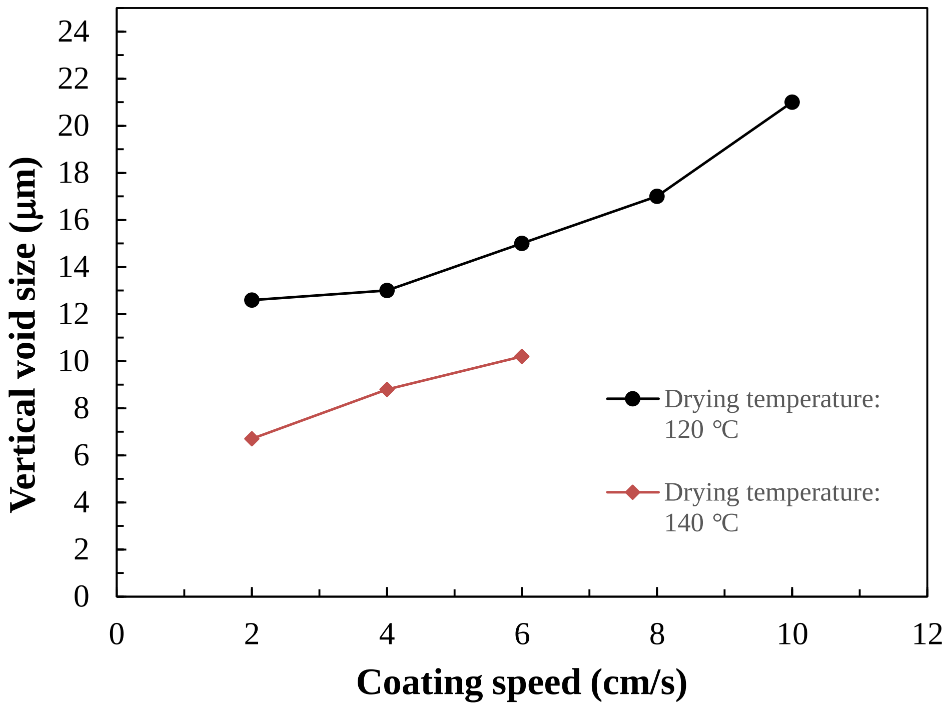

Figure S4. Correspondence between vertical void size and coating speed with drying temperature of 120 °C and 140 °C.

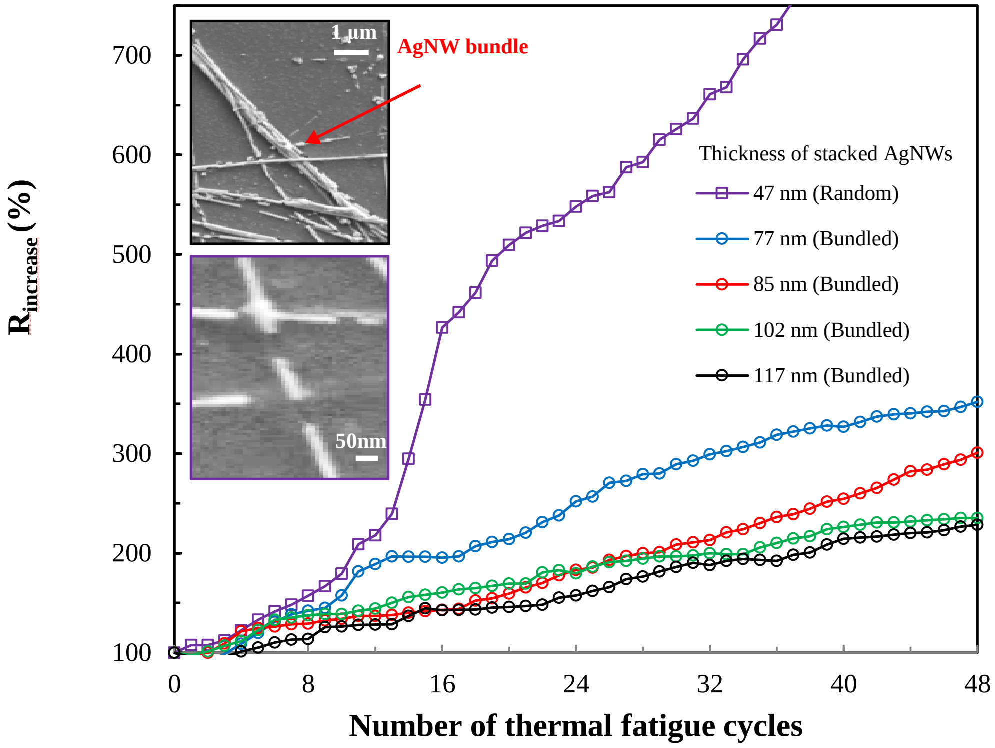

Figure S5. Thermal fatigue cycles for different AgNW electrodes. (Inset) SEM image of bundled AgNWs (upper) and randomly oriented AgNWs (lower) after thermal fatigue cycles.

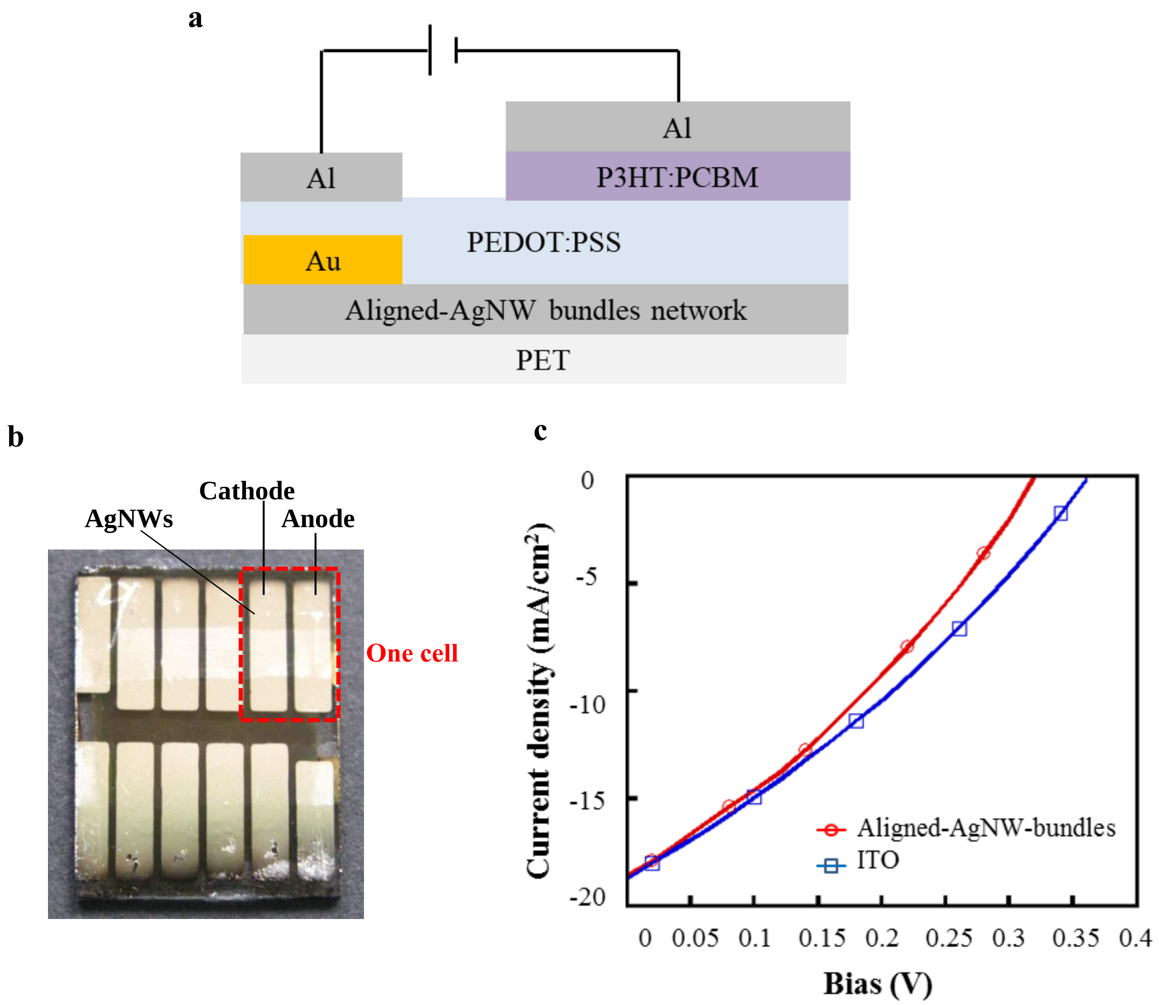

Figure S6. Comparison of ITO and AgNW-bundle networks in solar cells on PET films. (a) Schematic of the solar cell structure. (b) Picture of the fabricated solar cell. (c) Current density versus voltage for P3HT:PCBM solar cells using ITO and aligned AgNW-bundle networks.
